# Protein Adsorption Patterns and Analysis on IV Nanoemulsions—The Key Factor Determining the Organ Distribution

**DOI:** 10.3390/pharmaceutics5010036

**Published:** 2012-12-21

**Authors:** Cornelia M. Keck, Mirko Jansch, Rainer H. Müller

**Affiliations:** 1 Department of Pharmaceutics, Biopharmaceutics & NutriCosmetics, Freie Universität Berlin, Kelchstr. 31, 12169 Berlin, Germany; E-Mails: mirko.jansch@gmx.de (M.J.); nanoteam@gmx.com (R.H.M.); 2 Applied Pharmacy Division, University of Applied Sciences Kaiserslautern, Campus Pirmasens, Carl-Schurz-Str. 10-16, 66953 Pirmasens, Germany

**Keywords:** 2-D PAGE, 2-DE, two-dimensional polyacrylamide gel electrophoresis, nanoemulsions, plasma protein adsorption, drug targeting, nanoparticles

## Abstract

Intravenous nanoemulsions have been on the market for parenteral nutrition since the 1950s; meanwhile, they have also been used successfully for IV drug delivery. To be well tolerable, the emulsions should avoid uptake by the MPS cells of the body; for drug delivery, they should be target-specific. The organ distribution is determined by the proteins adsorbing them after injection from the blood (protein adsorption pattern), typically analyzed by two-dimensional polyacrylamide gel electrophoresis, 2-D PAGE. The article reviews the 2-D PAGE method, the analytical problems to be faced and the knowledge available on how the composition of emulsions affects the protein adsorption patterns, e.g., the composition of the oil phase, stabilizer layer and drug incorporation into the interface or oil core. Data were re-evaluated and compared, and the implications for the *in vivo* distribution are discussed. Major results are that the interfacial composition of the stabilizer layer is the main determining factor and that this composition can be modulated by simple processes. Drug incorporation affects the pattern depending on the localization of the drug (oil core *versus* interface). The data situation regarding *in vivo* effects is very limited; mainly, it has to be referred to in the *in vivo* data of polymeric nanoparticles. As a conclusion, determination of the protein adsorption patterns can accelerate IV nanoemulsion formulation development regarding optimized organ distribution and related pharmacokinetics.

## 1. Introduction

Realizing the concept of the “magic bullet” by Ehrlich is the goal in drug delivery [1,2]. Ideally the formulation should deliver most of the drug at its desired site of action, thus increasing therapy efficiency and minimizing undesired side effects. Many efforts were made during the last decades, leading to the first targeted formulations and commercial products. These products were not only developed for therapy, but also for diagnosis. The principle of action are antibodies, which are attached to the surface of liposomes to which the active ingredient is bound or incorporated. The antibodies bind to a specific antigen, leading to an increased active concentration at the target site, e.g., being sufficiently high for diagnostic purposes. An example is VesCan™ (Vestar Inc, San Dimas, California, USA), which is a tumor diagnostic agent [3]. Targeting was efficiently realized in diagnostics and therapy on a molecular level by attaching diagnostic molecules or radioactive isotopes (e.g., Indium 111 [4,5]) directly to site-specific antibodies. For example, targeting could be realized in anti-cancer treatment. An example is Ibritumomab tiuxetan (Zevalin^®^, Bayer Schering AG) for the treatment of B-cell non-Hodgkin’s lymphoma. It is a conjugate of the IgG1 antibody ibritumomab and the chelator tiuxetan, to which a radioactive isotope is added. The antibody specifically targets the conjugate to the CD20 antigen, which is only located on the surface of B-cells. By this, the radioactive isotope acts directly at the desired site of action, with less harm to healthy cells. However, the goal of targeting intravenously injected drug carriers for therapy is still unreached. The goal is considered as not yet being reached based on the following criteria:

1. no pharmaceutical products on the market for the therapy of patients;
2. despite the first successes in academic research, no “academically available” system is efficient/safe enough to be used for targeting in therapy.

There is a number of IV lipidic nanoparticulate carriers on the market for therapy. Examples are products based on the *o*/*w* nanoemulsions for parenteral nutrition, such as Intralipid^®^ and Lipofundin^®^. Products based on these emulsions mainly contain the drugs diazepam (Diazemuls^®^, Diazepam^®^-Lipuro, Stesolid^®^), propofol (Diprivan^®^, Popofol^®^-Lipuro 1%–2%, Propofol 1%–2%) CT Fresenius Emulsion, Propofol-ratiopharm 10 mg/mL–20 mg/mL), etomidate (Etomidat-Lipuro^®^) and dexamethasone (Lipotalon^®^). The other important IV lipidic carriers are liposomes, e.g., loaded with amphotericin B (AmBisome^®^), doxorubicin (Doxil^®^, Caelyx^®^, Myocet^®^) and daunorubicin (DaunoXome^®^). Incorporation of the drugs into liposomes solubilizes them and leads to a reduction of side effects. Examples are a decrease in nephrotoxicity in the case of amphotercin B and a reduced cardiotoxicity in the case of doxorubicin and daunorubicin. However, these emulsions and liposomes are actually not targeted systems. The effects are simply reached, e.g., by reducing the concentration of free drug in the blood (e.g., amphotericin B, anti-cancer agents), or at least at the injection site (e.g., diazepam).

A nice example of how efficient targeting can be is IV particulate targeting with polymeric nanoparticles loaded with the drug dalargin. However, it is still mainly on the level of academic research; presently attempts are being made by the German company Capsulution Pharma AG to develop a commercial product. Dalargin is a Leu-enkephalin analogue, known to interact with peripheral, but not central opiate receptors [6,7]. Hence, the administration of dalargin in solution does not lead to an analgesic effect, as the drug cannot pass the blood brain barrier (BBB). Kreuter *et al.* proved in *in vivo* studies that the administration of free drug does not lead to a nociceptive effect, but showed when dalargin was loaded onto polymeric nanoparticles, such an effect can be obtained. Hence, the particles targeted the drug across the BBB [7]. The mechanism of action is the binding of apolipoprotein E (Apo E) to the surface of the nanoparticles. Also, other drugs could be delivered to the brain using this concept. Examples are doxorubicin, loperamide, tubocurarine, the NMDA receptor antagonist MRZ 2/576 and kyotorphin [8,9,10]. Even though these nanoparticles are able to deliver drugs across the BBB, the percentage of the injected effective dose reaching the brain is relatively low. The mass of the brain represents only 1% of the body mass; hence, if 1% of the injected particles would reach the brain, this would mean that the BBB has no influence for these particles. However, based on published data, it can be assumed that it is generally below 1%. The reason for the loss is that prior to reaching the BBB, most of the loaded particles accumulate in the macrophages, mainly in the liver. Nevertheless, the results obtained by now are very promising, but to be used in therapy, the system still requires much further development.

The main hurdles for efficient targeting of all IV injected nanoparticulate delivery systems, including *o*/*w* emulsions, are:

1. overcoming the recognition of the injected particles as being foreign and their subsequent clearance by the macrophages of the mononuclear phagocytic system (MPS), mainly uptake by liver and spleen macrophages (up to 90%–95% of the injected dose);
2. and the lack of a sufficiently specific targeting moiety, which at the same time is not a too complex system to be realized over a foreseen period.

This article very briefly reviews the approaches to avoid the uptake into the MPS and the attempts for targeting. It focuses on the concept of “differential adsorption of blood proteins” for the targeting of IV carriers. A review of protein adsorption patterns analyzed on lipidic *o*/*w* nanoemulsion carriers is given and discussed regarding targeting optimization *in vivo*. This also includes targeting to the brain via Apo E.

### 1.1. Previous Strategies for IV Targeting

The aim in IV targeting is to design carriers the way that they preferentially accumulate at the desired site of action. To do this, the parameters being responsible for or causing this accumulation need to be known. In the middle of the 20th century, a very simplistic approach was taken. One tried to correlate the *in vivo* organ distribution (e.g., uptake by the MPS organs, such as the liver) to just one physico-chemical property of the injected particles (e.g., particle size or particle charge (zeta potential)). For example, it is described that large particles are cleared faster from the blood by the macrophages than small particles [11,12,13]. Positively charged particles are cleared faster than negative ones, the clearance being lowest for non-charged particles [14,15].

However, when changing the size of particles, at the same time, one will also change other physico-chemical parameters of the particle, e.g., total surface area, charge density on the surface or surface hydrophobicity. For example, polystyrene (PS) standard particles of different sizes are not identical in their surface properties. This is because different sizes are polymerized by using different polymerization conditions (e.g., various concentrations and types of catalyst for the reaction). This leads to differences in charge and surface hydrophobicity [13,16]. Therefore, measured *in vivo* effects of differently sized nanoparticles are not only related to just this single changed parameter (*i.e.*, size), but also to the change in other parameters occurring simultaneously when altering the particle size.

Awareness of this superimposed effect led, in the 1980s, to the concept of a “thorough characterization” of the particles in as many physico-chemical properties as possible. Efforts were mainly driven by S.S. Davis [17] and R.H. Müller [13]. Methods were established to quantify, for example, surface hydrophobicity, e.g., by performing adsorption studies with the dye Rose Bengal [13,18,19] or by using hydrophobic interaction chromatography (HIC) [13,20,21,22]. Also, measurements were performed to characterize the interaction with serum proteins by measuring the zeta potential in serum. The data allowed some explanations of the *in vivo* fate regarding the uptake by liver macrophages and avoidance of the MPS.

However, by this data, in many cases, no satisfying explanation or none at all could be given for the observed *in vivo* effects. The classical example is 60 nm polystyrene particles surface-modified (coated) by the adsorption of either poloxamine 908 or poloxamer 407. Both types of particles avoided the MPS, but the poloxamine 908 coated particles circulated in the blood [23,24], whereas the poloxamer 407 coated particles accumulated in the bone marrow [25]. Interestingly, the bone marrow accumulation was not immediate, as the particles circulated in the blood with an increasing accumulation over time, as seen from gamma scintigraphy images. After approximately four hours, a maximum accumulation was reached. Hence, there was obviously a time-dependent process, which promoted increasing uptake with time. The avoidance of MPS uptake could be explained by the physico-chemical characterization parameters of both of these particles: they possessed identical properties having a hydrophilic surface, low zeta potential in water, similar thick sterically stabilizing adsorption layers (around 12–14 nm) and yielded a low zeta potential in serum. All these parameters were previously identified as pre-requisites for avoidance of the MPS [13]. However, this could not explain why the physico-chemically similar particles were distributed differently in the body, blood circulation *versus* bone marrow accumulation.

As a next consequent step, considerations were made to develop characterization methods, which would allow us to identify the reasons for different accumulation profiles-despite similar or even identical measured physico-chemical properties. It was clear that particles can be identical in their measured properties, but still differ in their accumulation profiles. The interaction with blood proteins was identified to play a key role in this regard, because upon IV injection, blood proteins adsorb onto the surface of the particles. These proteins determine to which cells/tissues the particles are going, and thus determine the fate of the particles *in vivo*. Hence, due to differences in physico-chemical properties not accessible by the applied *in vitro* characterization methods (or being below their detection limit), different blood protein adsorptions patterns will result for different particles upon IV injection [26,27,28,29]. Therefore, it was logical to analyze the blood protein adsorption patterns of these particles and to correlate them to their respective organ distributions.

### 1.2. The Concept of Differential Protein Adsorption

Many previous studies investigated the adsorption of proteins onto particles. In most cases, only a single protein was studied (e.g., human serum albumin (HSA)) [30], or mixtures of two proteins, e.g., albumin and fibrinogen protein [31,32]. Studies with more than one protein are meaningful, because, in general, the presence of a second protein affects the adsorption behavior of the first protein and *vice versa*. However, these studies do not reflect the real situation in the blood after IV injection of particles. In the blood are more than a few thousand proteins [33], about a few hundred in higher concentrations. All these proteins compete for the adsorption to the nanoparticle surface. Therefore, the best approach and the only valid one for drawing useful conclusions to the organ distribution is the determination of the full protein adsorption patterns in the blood.

**Figure 1:**
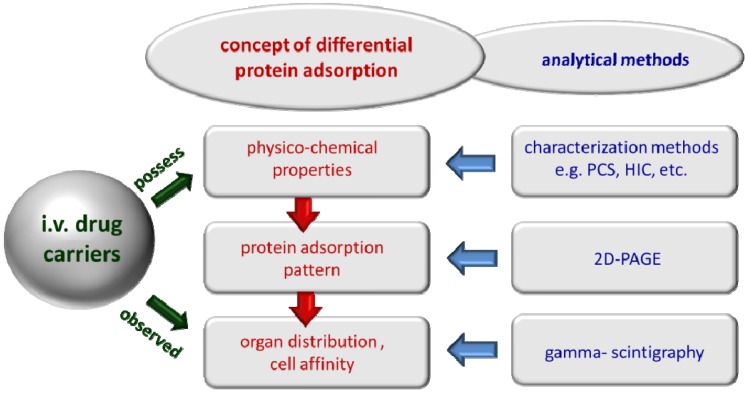
Concept of differential protein adsorption: the physico-chemical properties determine the composition of the protein adsorption pattern; the adsorbed proteins determine the subsequent organ distribution (modified after [24]).

Based on these considerations, the concept of differential protein adsorption was developed [27,28]. The blood proteins have different properties and will bind to different cells accessible from the blood. Therefore, the proteins that bind to a particle surface will determine to which cells, accessible from the blood, the particles will adhere. Due to the close contact to these cells and the increased concentration at this site, the particles are potentially taken up by these cells, e.g., by pinocytosis/endocytosis. Of course, the protein adsorption pattern acquired in the blood will depend on the physico-chemical properties of the particles. A correlation exists between physico-chemical properties, acquired adsorption patterns and resulting organ distribution (Figure 1). 

Within this concept, the correlation should be established, and then, this knowledge should be used to design target-specific IV carriers in a controlled way. To establish this correlation, quite a number of various IV injected nanoparticles with known organ distribution and surface properties were analyzed in the research group of R.H. Müller in Berlin regarding their adsorption patterns. The same targeting principle can be applied to IV *o*/*w* nanoemulsions. Their adsorption patterns—and how to modulate them—are reviewed in this publication.

Of course, in the case of biodegradable nanoparticles, the effects of biodegradation will overlap with the adsorption effects. Therefore, in many studies, non-biodegradable nanoparticles were used as model drug carriers. Many of them were especially synthesized by B.-P. Paulke, possessing tailor-made surface properties, e.g., the density of functional groups, the length of grafted polyethylene glycol (PEG) chains, the charge density and the surface hydrophobicity. Examples are different latex particles with sulphate and hydroxyl groups on the surface, e.g., latex with hydroxyl groups on a polystyrene surface, latex with hydroxyl groups on a poly (methacrylate) surface, latex with ammonium and hydroxyl groups on the surface and latex with ammonium and amidinium groups on the surface [34,35,36,37,38,39,40,41,42,43]. Besides these model particles, also the “real life” drug carriers were investigated. This includes various polymeric nanoparticles as potential drug carriers, magnetites for diagnostic purposes [37,44,45,46,47], as well as lipidic nano carriers. Under the polymeric nanoparticles were, e.g., polybutylcyanoacrylate (PBCA) [7,48,49,50,51,52], poly (lactic acid) (PLA), poly (lactic-co-glycolic acid) (PLGA) and poly (varepsilon-caprolactone) (PCL) [53,54,55]. 

The investigations on nano lipid carriers are the focus of this review. An advantage of these lipidic carriers are that they are very well tolerated by the body. In contrast to polymeric nanoparticles, they have an accepted regulatory status, e.g., parenteral *o*/*w* nanoemulsions or liposomes, and can be used in humans. Most of the polymers used in polymeric nanoparticle production possess no regulatory status or are only allowed for injection in microparticulate, but not nanoparticulate form (e.g., PLA/GA copolymers). They need a separate acceptance in nanoparticulate form, because the cytotoxicity of nanoparticles is different to microparticles (*cf.* increasing discussion about nanotoxicity). Per definition, nano lipid carriers are lipidic carriers in the nanometer range, where the matrix is either liquid (nanoemulsions) or solid (solid lipid nanoparticles (SLN) or nanostructured lipid carriers (NLC)). The nano lipid carriers covered in this article are *o*/*w* nanoemulsions for IV administration. Nanoemulsions were selected, because in contrast to the other particles, the *in vivo* studies available show excellent tolerability [56]. Therefore, nanoemulsions are not just “model carriers”, but are already used in humans and possess a high potential for future use in targeted drug delivery. Thus, the knowledge of parameters affecting the protein adsorption pattern is a powerful aid for further developments and controlled product optimization. Therefore, the data available up to now are reviewed in this article, and moreover, the data were re-evaluated and compared to each other. This should provide a clear summary of the influences being most crucial for the composition of the adsorption patterns determining the organ distribution and essential to be known for successful drug targeting.

### 1.3. Basic Considerations for Analysis of Protein Adsorption Patterns

Basically, the technique for protein analysis, two-dimensional gel electrophoresis (2-D PAGE, 2-DE) is well established, e.g., used also for diagnostic purposes in the clinic [57,58]. Nevertheless, analysis of the blood protein adsorption patterns is very complex and, thus, not trivial. The problem is the sample preparation. Potential artifacts, which can occur, require intensive validation and make the process very complex. This is the reason why only a few research groups around the world have a focus in this area [59,60,61,62,63,64]. This paragraph briefly touches on some of the critical parameters to give an impression of the complexity; the topic itself would rather require a review article on its own. Examples of critical parameters are:

1. choice of incubation medium;
2. species specific patterns;
3. ratio of particle suspension to incubation medium;
4. separation method;
5. washing medium and number of washing steps;
6. incubation time;
7. number of samples/repetitions.

The problem starts with the incubation medium for the particles. It can be either blood, plasma or serum. Ideally, blood should be used, but it is very difficult to separate the nanoparticles from the blood cells. In addition, the monocytes present in the blood start phagocytosing the particles, thus the particles disappear. *In vivo*, this happens only to a limited extent, because particles are taken up, e.g., within five minutes by the liver macrophages or accumulate somewhere else. Therefore, in general, plasma or serum is used instead of blood.

However, serum and plasma yield different patterns, because, e.g., serum is depleted from proteins of the complement system due to the coagulation process (e.g., lack of C3 and fibrinogen). The adsorption of a single protein depends on a composition of the mixture from which the adsorption takes place [65,66]. Moreover, in various studies with different types of particles, the adsorption of fibrinogen was demonstrated [37,67,68,69,70]. Many authors described the use of plasma as an incubation medium to be a reasonable compromise for the *in vitro* evaluation of colloidal drug carriers [39,46,67,71]. It is important, however, to bear in mind that plasma contains, additionally, compounds to inhibit coagulation, e.g., citrate or heparin, which can also interfere with the protein adsorption process. To minimize such effects and to obtain comparable results, one avoids the macromolecular heparins. Hence, very often, citrate plasma is used as an incubation medium.

The composition of the plasma proteins is different for each species. Therefore, the protein adsorption pattern of a particle will be different for each species. Due to this, in general, plasma from this animal species in which the *in vivo* studies are actually performed needs to be used for analysis. However, the question arises to which extend these patterns can be transferred to other animal species and, finally, to humans.

Also the ratio of particle suspension to incubation medium is important. In case the ratio is too high (too many particles, too large of a surface area), some high affinity and lowly concentrated proteins in the plasma or serum might be depleted. They would have been adsorbed in a higher amount if more protein would have been available. *In vivo*, one injects, for example, 1–5 mL particle suspension into humans, which corresponds to a volume of about 5–6 liters of blood (corresponding to approximately 3.5 L plasma). The protocol “NCL Method ITA-4” Version 1.1 [72] suggests to mix 1 mL plasma with 1 mL particle suspension (concentration 1 mg/mL = 0.1%). From our studies, reproducible patterns were obtained at a ratio of 3:1 plasma to suspension [48,73,74,75,76,77,78,79,80]. Please note, not the suspension volume, but the particle concentration and related surface area for adsorption are important. Typically, the particle concentration in our studies was in the range 10%–20% (*w*/*v*) when using the 3:1 ratio. In general, it is recommended to admix a particle suspension as highly as concentrated to plasma or serum to avoid too strong a dilution by the water of the particle suspension, to avoid reducing artificially the solubility of some proteins by the addition of water. Strictly speaking, for each nanoparticulate system, the correct ratio needs to be assessed, *i.e.*, the concentration of the sample above which the patterns do not change when more plasma is in the mixture (=exclusion of depletion).

Also, the separation method is crucial. Some of the currently used methods are centrifugation [76,78,81,82], chromatography [75,81,83,84] and separation by magnetic fields [44,85]. The separation method can affect the pattern. Even when incubating a sample for 5 min and then separating it, it will not represent the five minute pattern, because adsorption continues during the centrifugation process and during the time when handling the sample after incubation (the transfer of the sample into the centrifuge, the time in between the washing steps *etc.*). These times need to be kept strictly the same from one experiment to the next. And, here, the problem starts that somebody uses a previously described method, but gets different results (*cf.* emulsion data published by Harnisch [78,79] *versus* Schmidt [80] (*cf.* discussion below in Section 2.2.9). Harnisch specified only the centrifugation time and *g* number (1 h, 15,000*g*), but not the exact time plan of the different steps and processing times. 

Chromatography might lead to desorption effects at the end of the separation process. The particle sediment from centrifugation needs to be washed to remove excess plasma in the pellet. Normally, several washing steps need to be applied. However, the question is: which washing medium to take, distilled water or buffer? In the case of buffer—which buffer in which concentration? The basic problem is that the different washing media have different solubility properties for proteins compared to plasma. For example, immunoglobulin γ is less soluble in water. Washing with water leads to artificial adsorption of immunoglobulin from the plasma still remaining in the pellet onto the particle surface, which needs to be considered when interpreting the patterns.

Another question is the incubation time. Very often, five minutes are taken, because in many studies, the objective is to find drug carriers avoiding adsorption of opsonins (e.g., immunoglobulin γ, complement factors and fibrinogen) [63,86] and exhibiting ideally adsorption of dysopsonins (e.g., serum albumin) [87,88]. *In vivo*, opsonization and subsequent clearance of the particles by the liver macrophages takes place very fast, typically within 5 min up to 90% of the injected dose is taken up by the liver [13,89,90]. Therefore, it can be assumed that in case particles are recognized by the liver macrophages, the responsible proteins will be adsorbed within these five minutes and will be detectable after this time of incubation. However, this is different for particles adsorbing certain proteins only after longer contact times with the blood, e.g., the particles accumulating in the bone marrow (*cf.* Section 1.1.). In almost all cases, the protein adsorption patterns are time-dependent (time-dependent kinetics of protein adsorption) [91], which complicates analysis.

In the first adsorption stage, proteins with low affinity, but being present in high concentration, will adsorb. Then, they will be gradually displaced by proteins being low in concentration, but having a higher affinity to the particle surface [92,93]. Such time-dependent adsorption patterns are described, for example, for model polystyrene particles, which were surface-modified with poloxamer 407 and poloxamine 908 [91]. Thus, strictly speaking, for each carrier, full time-dependent adsorption patterns would need to be determined. This, of course, would increase the number of samples and the required time and costs tremendously. For an explanation—one adsorption pattern is obtained by one 2-D PAGE gel. For an optimal resolution, our 2-DE chambers are only filled with six, and not with 12, gels (more efficient cooling, higher reproducibility). To check the validity of the data, strictly speaking, three replicates (three gels per sample) would be needed. Assuming, now, 10 time points of incubation, this would require a total of 30 gels, plus reference standard gels. This set up is equivalent to filling about six chambers, which translates into an analysis time of about two weeks for one person and a cost of about €2000 just for the gels (one ready-made gel is about €60). This explains—apart from the complexity—why academic laboratories with limited budgets are not very active in this research area. To limit costs and time, selected incubation times are taken and—in most cases—only one gel per time point. However, this can only be done if the process was validated very carefully in regard to reproducibility, e.g., performing studies with three gels per time point for assessment of typical variation, *etc.*

In this regard, also, a special protocol for the analysis of the nanoemulsions was developed [78]. It is based on previously established protocols of the 2-D PAGE analysis of protein adsorption patterns on polymeric nanoparticles [67,81] and liposomes [83]. 

Briefly, the modified steps for the sample preparation by Harnisch and Müller are:

- incubating 2 mL of the emulsion in 6 mL citrate stabilized human plasma for 5 min at 37 °C;
- separating from excess plasma by centrifugation at 15,000*g* for 1h at 20 °C;
- washing three times with 0.05 M phosphate buffer, pH 7.4 and subsequent centrifugation (15,000*g* for 1h at 20 °C);
- solubilizing with sodium dodecyl sulfate (SDS) and dithioerythritol (DTE) for 5 min at 95 °C;
- cooling down to room temperature and incubation with a solution containing DTE (Dithioerythritol), CHAPS (3-(3-Cholamidopropyl)dimethylammonio-1-propansulfat), urea, Tris (Tris(hydroxymethyl)-aminomethan) and bromphenol blue, stirring and centrifuging 10–15 min at 12,000*g*;
- following steps according to the respective references given.

Special attention needs to be given to avoid oil residues, because of possible interference of lipids with proteins and the gel matrix [78] and the use of thiourea, which has been found to further improve solubilization [94,95]. Changes in the protein adsorption pattern caused by altered surface properties of the emulsion (*i.e.*, adsorbed poloxamer 407) were nicely detectable by using this optimized protocol [78]. 

## 2. Protein Adsorption Patterns of Different IV Nanoemulsions

### 2.1. General Aspects

Lecithin stabilized *o*/*w* nanoemulsions are used in parenteral nutrition since the 1950s [96,97,98,99]. The oil phase (10% or 20%) is, in most cases, composed of long chain triglycerides (LCT) alone or is a mixture with medium chain triglycerides (MCT); also used are fish oil and safflower oil. They have an excellent record of tolerability, even in the large quantities used for parenteral nutrition (e.g., 500 mL/day). From this, they are an ideal carrier for poorly water soluble, but lipophilic, drugs, such as diazepam or propofol. 

A potential side effect of IV injected emulsions is the impairment of the MPS due to too excessive of an uptake by the MPS, mainly liver and spleen macrophages. Ideally, the macrophage uptake should be low, so that the emulsion droplets can be metabolized for nutrition, e.g., by the lipases of the endothelia in the blood capillaries. Impairment of the MPS can be tested *in vivo* by infusion of a parenteral emulsion and subsequent injection of a radioactive test colloid with a known high uptake by the liver macrophages. In case the emulsion droplets have been heavily taken up by the liver macrophages, accumulation of the radioactive test colloid in the liver will be reduced. The extent of reduction is a measure for the impairment of the MPS [100]. Complementary to this, the protein adsorption patterns can be investigated. In general, MPS uptake of these emulsions is low, e.g., due to the hydrophilic nature of the lecithin surface. Analysis of the patterns can therefore provide information on how patterns need to look for the avoidance of or, at least, the minimized uptake by the MPS. In general, the adsorption of opsonins (e.g., fibrinogen) enhances MPS uptake, and the adsorption of dysopsonines (e.g., albumin) minimizes MPS uptake. However, up to today, no all over solution for the interplay between physico-chemical properties and the *in vivo* fate can be given. Many studies have been performed by now, mainly to identify the parameters that affect the protein adsorption most. These data are reviewed in the sections below, with the aim of providing an overall picture. 

### 2.2. Comparison of Adsorption Patterns of Different Nanoemulsions and Influencing Parameters

#### 2.2.1. Adsorption Patterns of 20% Emulsions Stabilized with Lecithin from Different Manufactures

Commercially available emulsions for parenteral nutrition of intensive care patients can differ in their distribution in the body [101,102]. Preferentially, they should show a low uptake by the MPS cells, especially the liver, to avoid impairment. Macrophages full of oil droplets cannot phagocytose bacteria and viruses from the blood stream anymore. Therefore, the manufacturers emphasize low MPS impairment for their products. Different nanoemulsions, principally possessing the same or a very similar composition, are available from different manufactures. However, each manufacturer has its own specific lecithin or lecithin fraction and details of the exact composition are not accessible. Therefore, the interesting question was if the adsorption patterns would differ due to variations in the lecithin composition or if the phospholipid as a structural element dominated the pattern.

In one study, four commercial *o*/*w* emulsions were investigated [103]. In detail: Lipofundin MCT 20% (B. Braun Melsungen AG, Melsungen, Germany), Intralipid 20% (Pharmacia AB, Stockholm, Sweden), Abbolipid 20% (Abbott, Wiesbaden, Germany) and Schwalipid 20% were compared to each other. For all investigated fat emulsions, the amount of stabilizer (lecithin) was about 1.2%, and the particle size distribution of all these emulsions differed just slightly (the mean diameter was about 250 nm). Hence, also, the size of the surface area in the analysis was practically identical. Almost no differences in the amounts of the major proteins, namely apolipoproteins A-I, A-IV, C-II, C-III and immunoglobulin D, adsorbed were found within the formulations. On average, the percentage of the overall detected amount of protein on the emulsions was 22.8%, 19.8%, 14.2%, 1.4% and 28.9% for A-I, A-IV, C-II, C-III and immunoglobulin D, respectively. The variations were ±1.6, ±0.3, ±0.7, ±0.3 and ±1.4 for A-I, A-IV, C-II, C-III and immunoglobulin D, respectively. Hence, the obtained plasma protein adsorption patterns were very similar; only slight differences were detected for the amount of apolipoprotein A-I. The conclusion from this study is that no significant difference was detected for the four emulsions investigated. Obviously, slight variations in the lecithin composition have no or little effect on the adsorption pattern, so the phospholipid structure dominates-independent of the slight compositional differences (e.g., in fatty acids or the type of phospholipid).

If organ distributions of such emulsions are different, it is not possible to explain the differences solely by comparing qualitatively and quantitatively the protein patterns obtained by 2-D PAGE analysis. A possible reason could be a difference in the conformation of certain proteins adsorbed on the particle surface. This has been emphasized for polymeric particles [104] and might occur for emulsions, too. Strictly speaking: for a full picture, it would also be necessary to know the conformation of the proteins. However, the conformation is not accessible analytically in such a manifold mixture of proteins in the adsorption layer. An additional aspect, which cannot be detected, is the degradation of the oil droplets, which will only take part *in vivo*, but not in the *in vitro* 2D PAGE analysis. The degradation will change the surface properties, and thus the adsorption pattern, over time. Depending on the composition (e.g., LCT *versus* MCT oil), this will occur differently for the different emulsions, thus leading to different organ distributions.

#### 2.2.2. Adsorption Patterns of Different Oil Compositions: LCT *vs.* LCT/MCT

**Figure 2:**
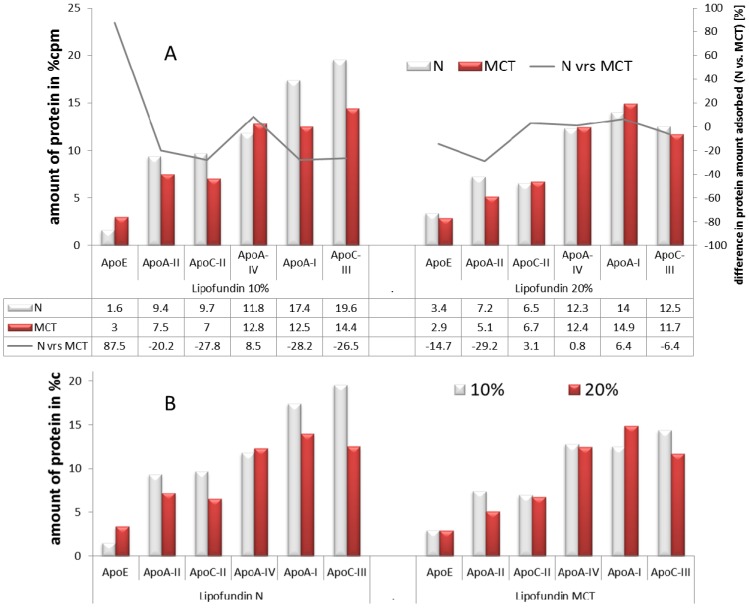
Influence of oil composition on protein adsorption patterns on adsorbed protein type and amount (as a percentage of the overall detected amount of protein, after [105]); (**A**) MCT *vs.* N for 10% oil content (left) and 20% oil content (right) and the difference in the amount of protein adsorbed given in %;(**B**) 10% *vs.* 20% oil content for Lipofundin N (left) and Lipofundin MCT (right10%).

Investigated were Lipofundin MCT 10% *versus* Lipofundin N 10% and Lipofundin MCT 20% *versus* Lipofundin N 20% [105]. Lipofundin N consists of soy oil (LCT—long chain triglycerides), Lipofundin MCT is a mixture of 50:50 LCT and MCT. Medium chain triglycerides (MCT) are reported to lead to a smaller impairment of the MPS than long chain triglycerides (LCT) [106]. The MCT oils are faster metabolized than LCT; this is claimed to reduce fatty metamorphosis of the liver [107]. In this protein adsorption study, all the investigated emulsions are stabilized with lecithin, but soy lecithin in the case of Lipofundin N and egg lecithin in the case of the MCT emulsions. Based on previous studies (*cf.* Section 2.2.1), the authors assumed that differences in lecithin are of no or little effect. Thus, potential differences in the adsorption patterns should result from differences in the oil composition. The 2-D PAGE gels (*cf.* supplementary data) show no qualitative differences; hence, similar types of proteins are absorbed. In Table S2, (left columns), the amounts of apolipoproteins as a percentage of the overall detected amount of protein are shown. It might be interesting that the amount of Apo E is nearly doubled on Lipofundin MCT 10%, and, in contrast, the apolipoproteins A-I, C-II and C-III are distinctly reduced. Also, the 2-D PAGE gels obtained from Lipofundin N 20% and Lipofundin MCT 20% show no differences in the quality of proteins that can be observed (*cf.* supplementary data). The dominating adsorbed proteins are, again, apolipoproteins. A quantitative summery is given in Figure 2A. This time, there was no major difference in Apo E adsorption detectable; both emulsions showed about 3% ApoE adsorption. The other apolipoproteins are rather similar. It seems, as with a higher concentration of the lipid component of the emulsion, the differences in the adsorption of the plasma proteins are getting less. Another point to keep in mind is that the 10% emulsions contains 0.8% Lecithin, while the 20% emulsions contains 1.2%, meaning the amount of lipid is doubled, but not the amount of emulsifier. Lecithin can form hydrated gel-like layers around oil droplets (emulsifier gel), forming a quasi third microscopic phase in the emulsion. The covering density on the droplet surface and the thickness of the lecithin gel phase might change with the amount of lecithin present in an emulsion and the ratio of lecithin to oil/droplet surface. As a summary, the composition of the oil core seems to affect the adsorption patterns, but only to a limited amount. However, also, the ratio of oil to lecithin might play a role, which is different in the 10% and 20% emulsions. 

#### 2.2.3. Adsorption Patterns of Different Oil Concentrations: 10% *vs.* 20%

To assess the influence of the lipid concentration, the data of the emulsion systems from above were plotted in separate graphs, e.g., emulsions having the same oil (LCT or MCT/LCT mixture) but two different oil concentrations were compared between each other [105]. The results are shown in Figure 2B. 

For both types of lipid, there is a small tendency for less protein absorption with an increase in lipid content. This seems to be more pronounced for the long chain triglycerides (Lipofundin N) than for the medium chain triglycerides (Lipofundin MCT). The most pronounced effect was observed for Apolipoprotein C III, known for being responsible for the inhibition of the liver uptake of triglycerides. Hence, the higher the amount, the slower will be the uptake of the lipid into the liver and *vice versa*. The presence of Apolipoprotein E (Apo E) is important for the metabolism of triglycerides, whereas less Apo E leads to increased levels of triglycerides in the blood. In the case of the Lipofundin N samples, Apo E was increased almost two-fold when the lipid phase was increased. In conclusion, the data indicate a lipoprotein-related enhanced uptake of triglycerides with an increased amount of the lipid phase. This effect was only observed for the long chain triglycerides, but not for the emulsions containing medium chain triglycerides. The MCT emulsions had a similar high Apo E level for both concentrations. Nevertheless, when comparing the emulsions, it has also to be kept in mind that the droplet sizes can be different. Often, 20% emulsions are larger in size than 10% emulsions, due to the production process. In the investigations by Schmidt (2002), the emulsions were typically in the range of 250–270 nm, only the 20% Lipofundin N emulsion was about 350 nm. The droplet surface to lecithin ratio is therefore quite similar for the MCT and the N 10% emulsions and smaller for the Lipofundin N 20%. 

#### 2.2.4. Effect of Type of Oil Phase on Protein Adsorption

So called “structured lipids” (SLs) are new chemical entities as alternatives to LCT in emulsions for parenteral nutrition. They are made by enzymatic transesterification of fatty acids in the 1,3 positions of the triglyceride [108]. The exchange of the long chain fatty acids in position one and three with short chain fatty acids lead to triglycerides with of a composition Short-Long-Short (SLS) fatty acids at the glycerol back bone and the respective exchange with medium chain fatty acids to MLM SLs, whereas LLL contains long fatty acids only (e.g., pure soy bean oil) (structures of SLs *cf.* supplementary data). 

**Figure 3:**
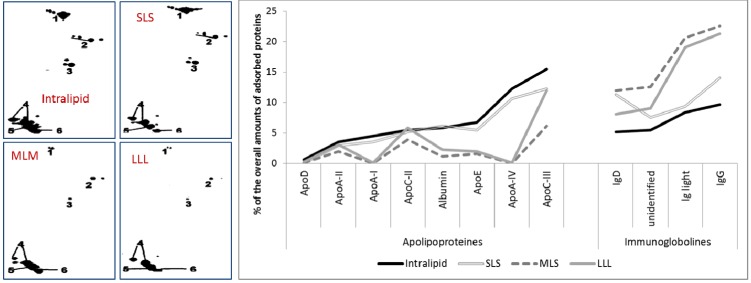
Adsorption of apolipoproteins on 10% Intralipid-, SLS-, MLM- and LLL-emulsions. **Left**: Close-ups of the bottom left part of the 2-D PAGE gels. Isoelectric point pI 4.4 to 6.0 (from left to right, not linear), molecular weight (MW) 6–46 kDa (from bottom to top, not linear). Protein spot number: (1) ApoA-IV, (2) ApoE, (3) ApoA-I, (4) ApoC-III, (5) ApoC-II, (6) ApoA-II. **Right**: type and amount of proteins adsorbed onto the different emulsion (modified after [108]).

The overall composition of the emulsions was: 10% lipid phase, 1.2% Lipoid E80 as an emulsifier and 2.1% glycerol. The average particle size was: 265 nm (Intralipid), 349 nm (LLL), 407 nm (MLM) and 306 nm (SLS). It was investigated if the exchange of the oil phase from vegetable oils by these SLs affects the protein adsorption patterns [108]. In Figure 3 (left), the apolipoprotein adsorption on the different types of emulsions are shown. Clearly, less protein was adsorbed on the MLM and LLL emulsions. The total apolipoproteins amounts adsorbed were 48.3% (Intralipid), 40.1% (SLS), 13.4% (MLM) and 22.2% (LLL), respectively. The individual amounts of the main adsorbed apolipoproteins and other main adsorbed proteins are summed up in Figure 3 (right). 

Very similar adsorption patterns were obtained for the SLS emulsion and Intralipid and for the MLS and LLL emulsion. The main difference is that SLS and Intralipid show higher amounts of adsorbed apolipoproteins and less immunoglobulins and other proteins when compared to MLM and LLL. Almost no difference was found for the apolipoproteins ApoA-II and ApoC-II. The largest differences within the two groups were found for the amounts of adsorbed ApoA-I and ApoA-IV, followed by ApoE and albumin. Hence, the amount of dysopsonines was much higher for SLS and Intralipid, and the amount of opsonines (e.g., immunoglobulins) was lower than for MLM and LLL. These findings might explain the *in vivo* difference in elimination found in another study, where the SLS emulsion based on structured lipids with short-chain fatty acids in the 1,3-positions was removed more slowly from the general blood circulation compared to emulsions based on lipids with long-chain fatty acids in the 1,3-positions (LLL) and MLM [108].

#### 2.2.5. Effect of Stabilizer Composition on Adsorption Patterns

**Figure 4:**
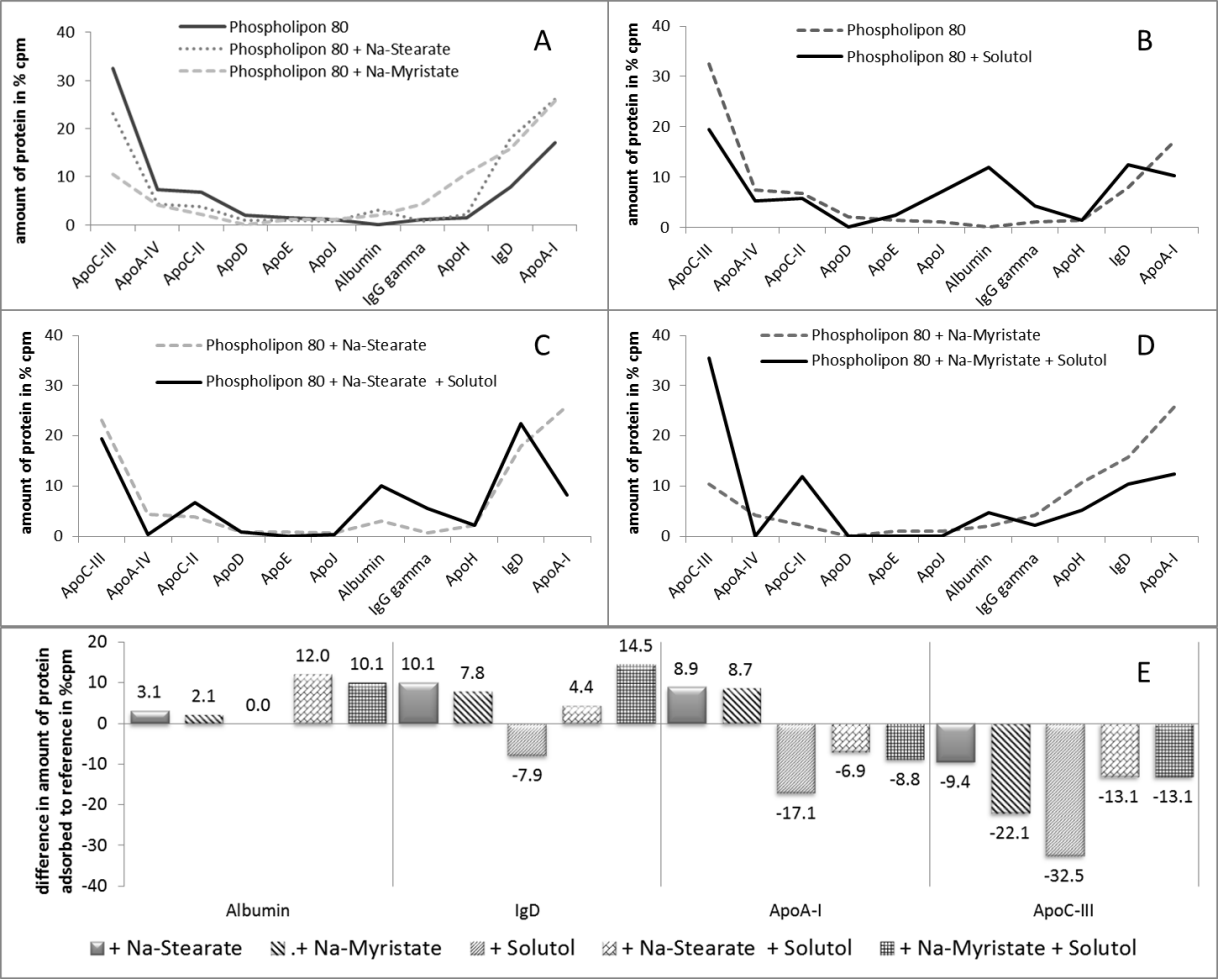
Type and amount of adsorbed plasma proteins on oil-in-water emulsions with different compositions of stabilizing agents. (**A**) Influence of the fatty acids sodium stearate and myristate in the composition of a Phospholipon 80 stabilized emulsion on the adsorption pattern;(**B**–**D**) Influence of sodium stearate and myristate in the composition of a Phospholipon 80/Solutol stabilized emulsion on the adsorption pattern (modified after [110]);(**E**) Difference of amount of adsorbed proteins due to different stabilizer composition compared to the standard stabilized with Phospholipon only.

The type and amount of adsorbed plasma proteins can be affected by two parts of the emulsion droplets: first, by the inner core—consisting of at least one type of triglyceride and potentially an active compound (drug); and second, by the outer stabilizing emulsifier layer [109]. To investigate the influence of the emulsifier on the adsorption pattern, Harnisch *et al.* investigated different emulsifier blends [110]. Briefly, the effect on fatty acids with different chain lengths (*C*14 and *C*18, *i.e.*, Na-Myristate and Na-Stearate, respectively) and a PEG containing stabilizer (Solutol) and mixtures of these stabilizers in comparison to a lecithin stabilized emulsion were investigated. For detailed information of the formulations investigated, *cf.* supplementary data. The results of this study are depicted in Figure 4. 

The addition of fatty acid salts, sodium stearate and myristate changes the adsorption patterns of the original emulsion stabilized with phospholipon alone. Three cases where observed. For one group of the proteins, a decreased amount of protein upon the addition of the fatty acids was observed (e.g., ApoC-III, ApoA-IV, ApoC-II; Figure 4A-left). For other proteins, no changes were observed (e.g., ApoD, ApoE, ApoJ, IgG gamma; Figure 4A-middle), whereas for a third group of proteins, an increase was observed (albumin, IgD, ApoA-I; Figure 4A-right). In the case of Ig G and Apo H, an increase was only observed upon the addition of the medium chain length fatty acid myristate, but not for the long chain acid stearate. Both Ig G and Apo H have manifold functions, thus an estimation effect that could result due to these differences cannot be given at this point.

The changes of the adsorbed proteins and also the effect of the two different fatty acids changed when a co-emulsifier (Solutol) was introduced in the systems. Solutol is an PEG containing non-ionic stabilizer. As PEGylation can cause a socalled stealth effect, where MPS uptake is circumvented, it was very interesting to investigate the changes upon the addition of Solutol (Figure 4B–D). 

Introducing PEG chains to the stabilizer layer led to an increase in the amount of albumin adsorbed. Albumin is a known dysopsonin; hence, less recognition by the MPS can be expected. The second effect observed was the increased amount of adsorbed ApoC-II when compared to the systems without Solutol. Apo C-II is known to activate the lipoprotein lipase and, thus, plays a key role in lipid metabolism. However, Apo C-II was reduced upon the addition of fatty acids (*cf.*Figure 4A); Hence, the addition of Solutol could reverse the Apo C-II reducing effects of the fatty acids. To investigate the all over effect of the different stabilizers on the adsorption pattern, the differences in the amount of adsorbed proteins were calculated in comparison to the original formulation containing only lecithin as a stabilizer.

Figure 4E shows the results of the proteins with major changes upon the addition of fatty acids and/or a non-ionic surfactant (e.g., albumin, IgD, ApoA-1 and Apo C-III). Upon the addition of Solutol, no changes were observed for albumin. A slight increase was observed upon the addition of the fatty acids, but the combination of Solutol and fatty acids further increased the amount of the adsorbed albumin by a factor of four to five. The amount of Ig G and Apo A-1 was increased when fatty acids were present, but was decreased when Solutol was present. In the case of Ig G, the addition of Solutol to the fatty acids did not lead to a decrease, whereas it decreased for Apo A-1. For Apo C-III for all formulations, decreased amounts were detected. This nicely shows how strongly and how differently changes in the composition of the stabilizer layer affect the protein adsorption patterns. In summary, the composition of the stabilizer obviously dominates the protein pattern of an emulsion, as the pattern is less affected by changes of the oil composition inside of the droplets. Changes in the stabilizer or the addition of a co-emulsifier can, therefore, be expected to change the organ distribution of IV emulsions.

#### 2.2.6. Surface Modification of Lipofundin Emulsions for Drug Targeting

In the previous study, the change of the stabilizer composition was obtained by producing different formulations. Of course, nanoemulsions can easily be produced with different stabilizers by applying high pressure homogenization. Stable emulsions can be obtained using stabilizers e.g., Tween 80, poloxamer 188 or poloxamine 908. By replacing the stabilizer, the adsorption pattern can be changed. Jeppson and Rößner reported already in the 1970s a reduced liver uptake of poloxamer 188 stabilized emulsions [111].

However, another possible way to change the surface properties of emulsions is the modification of commercially available parenteral emulsions by the simple addition of surfactants or stabilizers, followed by an incubation time [78]. The method is very elegant and highly efficient, because ready produced emulsion can be used. The additional stabilizer (or a mixture of different stabilizers) is admixed to a ready-made emulsion. After addition—according to the Nernst partitioning coefficient—the added surfactant partitions between the water phase and the stabilizer layer. Based on the partitioning coefficients, a mixed surfactant layer is formed with altered physico-chemical properties and a subsequently altered protein adsorption pattern and related organ distribution. This is a smart approach to create targeted nanoemulsions.

**Figure 5:**
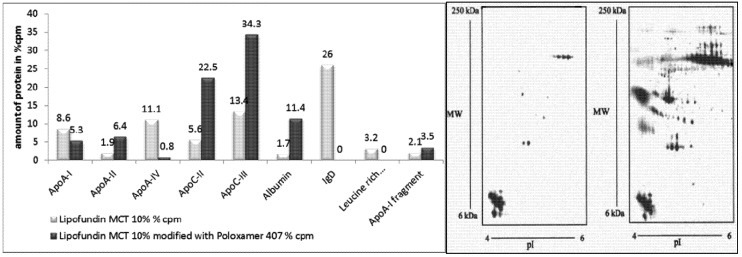
Modification of the adsorption pattern by incubation. Left: Total amount of proteins (cpm) and number of protein spots on Lipofundin MCT 10% emulsions, non-modified and surface-modified with poloxamer 407, and after artificial contamination of the protein solution with emulsion. Right: Close-ups from non-modified Lipofundin MCT 10% emulsion (left) and from Lipofundin MCT 10% emulsion modified with poloxamer 407 (right). The close-ups cover an isoelectric point from 4 to 6 and a molecular mass of 6–250 kDa (non-linear). The first dimension was carried out using IPG strips (after [78]).

In a study by Harnisch *et al.*, Lipofundin MCT 10% and an equal volume of 2% poloxamer 407 solution were mixed, incubated and the resulting adsorption pattern analyzed. The modification led to a very pronounced change in the patterns (Figure 5). There was a distinct reduction in the amount of overall detected protein from 1387 cpm to 472 cpm (arbitrary unit), the number of detectable protein spots decreased from 407 to 58, and, most remarkably, a relative enrichment of Apo C-II and Apo C-III was obtained. These are apolipoproteins being held responsible for accumulation of poloxamer 407-stabilized 60 nm polystyrene particles in the bone marrow (*i.e.*, the ratio of II to III).

#### 2.2.7. Influence of Surface Charge

Surface modified cationic emulsions elicited *in vivo* a much longer circulation time within the bloodstream than anionic emulsions (e.g., Intralipid) and enhanced drug uptake to a specific side of action [112,113]. Korner *et al.* reported that the long circulating effect of the cationic emulsions is based on the composition and conformation of the used emulsifier film at the *o*/*w* interface [114]. A study from Tamilvanan *et al.* investigated the effects of a surface modified cationic emulsion on the *in vitro* plasma protein adsorption pattern [115]. Four different formulations (two anionic and two cationic emulsions) and the commercially available anionic emulsion Lipofundin MCT 10% were used to investigate and compare the protein adsorption patterns onto emulsion droplets. The standard composition consisted of MCT (8.5% *w*/*w*), Lipoid E-80 (1.2% *w*/*w*), α-tocopherol (0.02% *w*/*w*), poloxamer 188 (2.0% *w*/*w*), glycerol (2.25% *w*/*w*) and bi-distilled water (to 100% *w*/*w*) with additional stearylamine (0.3% *w*/*w*) or oleylamine (0.3% *w*/*w*) for the two cationic formulations and oleic acid (2.83% *w*/*w*) or deoxycholic acid (0.5% *w*/*w*) for the two anionic formulations. The physical characterization of these nanoemulsions and the results of the 2-D PAGE analysis are shown in Table 1.

**Table 1:** Physical characterization of the nanoemulsions (* *n* =3) and comparison of values (volume%) of adsorbed plasma proteins on surfaces of developed anionic and cationic emulsions and Lipofundin MCT 10% (after [115]).

Formulations	Anionic emulsions prepared based on		Cationic emulsions prepared based on		Lipofundin MCT 10%
	Oleic acid	deoxycholic acid	stearylamine	oleylamine	
*A: Physical characterization of the nanoemulsions*					
particle size * (nm ± SD)	205 ± 52	192 ± 48	183 ± 30	167 ± 50	250 ± 14
zeta potential * (mV ± SD)	(−36.1) ± 3.6	(−34.4) ± 0.8	47.8 ± 0.9	37.2 ± 2.4	(−36.0) ± 0.3
*B: Volume percentage of detected plasma proteins after their adsorption onto particles*					
Albumin	0.0	2.9	2.7	2.5	2.0
ApoA-I	25.0	9.7	33.2	30.5	9.7
ApoA-II	2.5	6.8	6.1	4.8	9.2
ApoA-IV	4.6	36.9	6.7	4.5	10.5
ApoC-II	1.8	12.0	6.2	4.5	5.6
ApoC-IIII	4.5	14.4	8.6	7.9	10.6
ApoE	0.0	1.9	1.5	1.2	2.9
ApoJ	0.0	0.4	0.8	0.8	0.5
Ig-gamma-chain	2.2	0.0	0.9	1.2	8.1
Ig-light chain	6.3	1.3	1.5	3.0	0.0
total	46.9	86.3	68.2	60.9	59.1

In general, the presence of poloxamer 188, a steric stabilizer, seemed to shield all of the tested emulsions from the adsorption of larger proteins, e.g., immunoglobulins, fibrinogen, *etc.* Smaller proteins, such as albumin and apolipoproteins, were almost completely present in the 2-D PAGE pattern. The cationic formulations, as well as the anionic oleic acid emulsion, adsorbed three-times more Apo A-I compared to the marketed Lipofundin MCT 10% and the deoxycholic acid-based anionic emulsion (Table 1). Furthermore, the Apo A-I/Apo A-IV ratio can be an explanation for the previously observed prolonged plasma circulating times of the cationic emulsions compared to the deoxycholic acid-based anionic emulsion. The ratio is above five for both cationic emulsions, one for Lipofundin and about 0.26 for the deoxycholic acid-based anionic emulsion. The other anionic formulation containing oleic acid has shown a similar adsorption pattern like the cationic formulations, which indicates the complexity of differential protein adsorption. Having a similar surfaces charge does not automatically lead to the same adsorption of proteins when using different stabilizers (deoxycholic or oleic acid). Nevertheless, deoxycholic acid possesses a few pharmacological properties, e.g., immunostimulant and healing processes [116,117,118]. The cause is thought to be the activation of macrophages. Hence, by these data, the fast recognition by macrophages might be explained by the large amount of adsorbed Apo A-IV.

In addition, these data show nicely that the previous approaches, by just looking at one physico-chemical parameter, e.g., particle charge, are too simplistic. A consideration of as many as possible accessible physico-chemical parameters is necessary. The resulting adsorption patterns are a result of many superimposing surface properties, in this case, the charge and presence of functional groups/different molecular structures and related changes in surface hydrophobicity.

#### 2.2.8. Effects of Drug Incorporation

##### 2.2.8.1. Effect of Amphotericin B

**Figure 6:**
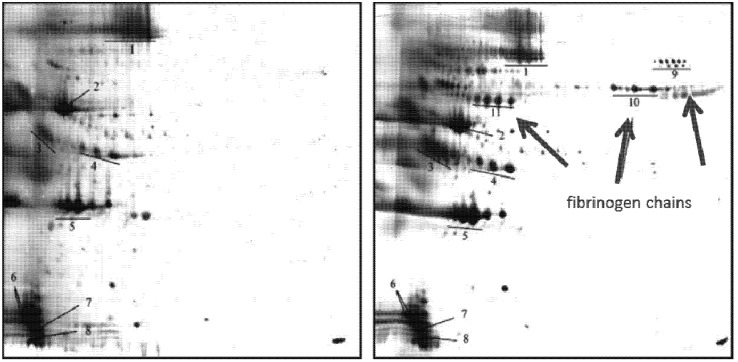
Comparison of silver stained gels of Lipofundin N 20% (**left**) and the amphotericin B-containing emulsion (**right**). Arrows mark the additional adsorbed fibrinogen chains. Protein spot number: (1) Albumin, (2) ApoA-IV, (3) ApoJ, (4) ApoE, (5) ApoA-I, (6) ApoC-III, (7) ApoA-II, (8) ApoC-II, (9) Fibrinogen-α-chain, (10) Fibrinogen-β-chain, (11) Fibrinogen-γ-chain (modified after [80]).

The effects of incorporation of amphotericin B and propofol on the adsorption patterns were investigated. Amphotericin B was incorporated in the commercial fat emulsion Lipofundin N 20% applying the SolEmuls process [119,120]. The drug loading was only 1 mg/mL, but it changed the adsorption pattern significantly (Figure 6; for quantitative data *cf.* supplementary data) [80]. The amounts of Apo A-IV, C-III and albumin were distinctly reduced. On the drug-loaded emulsion, different fibrinogen chains adsorbed. Apolipoproteins are classified as dysopsonins, reducing phagocytosis, whereas fibrinogen, as opsonin, enhances phagocytosis. Based on this, a different *in vivo* distribution can be expected for the drug-loaded emulsion. This is in line with *in vivo* studies of lipid formulations of amphotericin B showing liver and spleen accumulation (and reduced kidney concentration responsible for the reduction of nephrotoxicity) [121].

##### 2.2.8.2. Effect of Propofol

**Figure 7:**
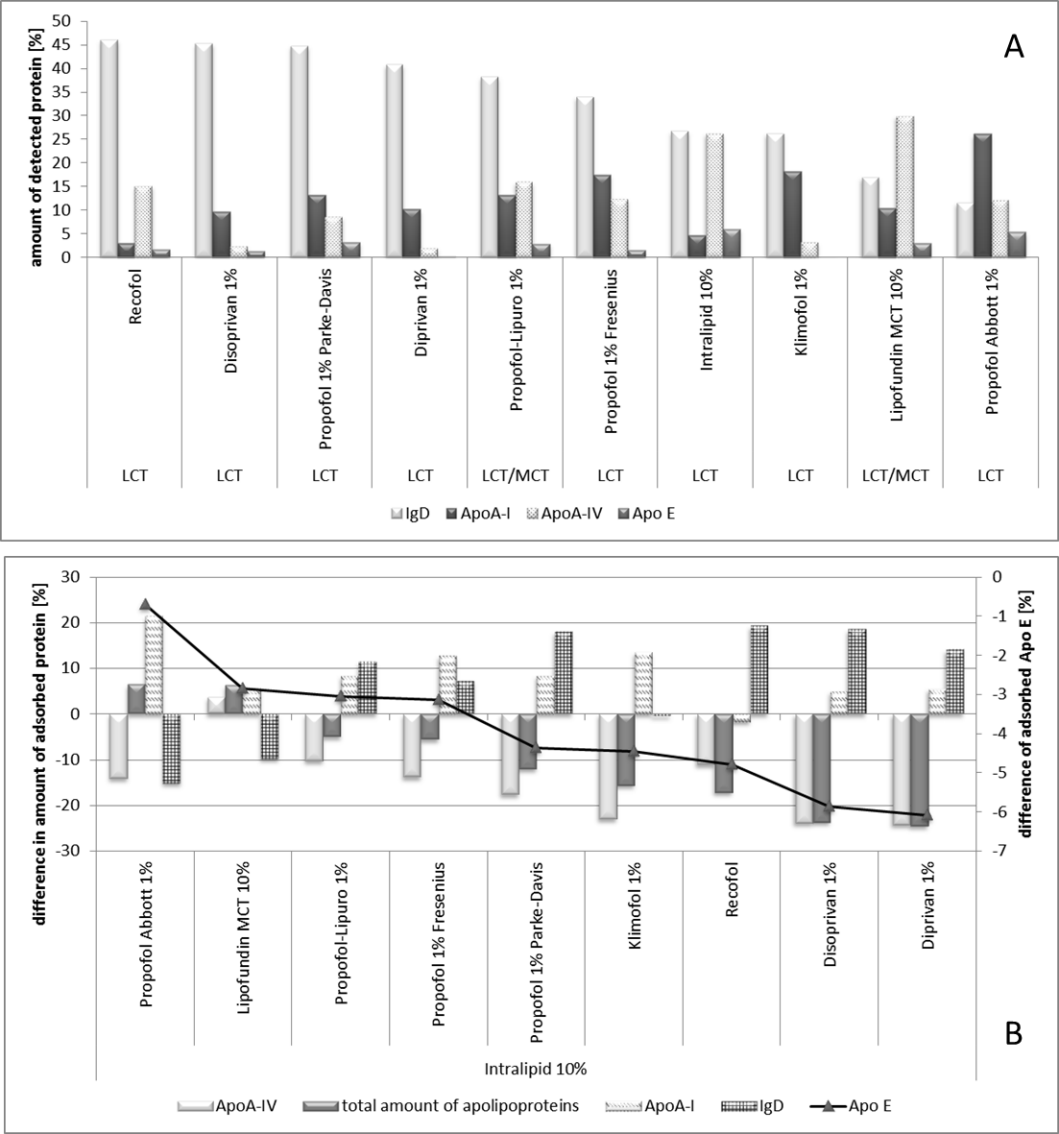
(**A**) Comparison of major proteins detected on the different propofol emulsions and the reference emulsions from which the propofol emulsions were derived. Protein amounts are given as a percentage of the total amount of protein detected on the 2-DE gels;(**B**) shown as differences in the amounts of adsorbed proteins in comparison to the original IV emulsion (Modified after [77]).

In the propofol study, eight different marketed propofol emulsions were investigated and compared with the respective emulsion base Lipofundin MCT 10% and Intralipid [77]. The investigated emulsions were all in the size range of about 200–259 nm (mean diameter photon correlation spectroscopy (PCS)). Figure 7 shows the results. 

In contrast to the amphotericin B-loaded emulsion, no adsorption of fibrinogens was detected. An explanation is the different localization of the two drugs. Amphotericin B is a more amphiphilic drug and, thus, it localizes preferentially in the interface (*i.e.*, within the stabilizer layer). Therefore, changing the physico-chemical properties of the interface and, subsequently, the adsorption pattern. Propofol is highly lipophilic and is, therefore, more located in the oil droplet. Still, all propofol emulsions led to different adsorption patterns (Figure 7A). To enable a better comparison, the results were calculated as differences in the amounts of adsorbed proteins in comparison to the original IV emulsion, respectively (being either Intralipid or Lipofundin, Figure 7B).

Propofol Abbott showed the most significant differences to the other emulsions investigated, but the least differences to the drug free base Lipofundin MCT. This effect cannot be explained. However, it should be noted that the content of free propofol in the water phase was different for the investigated emulsions, which might affect the surface properties of the droplets. However, for all other emulsions, a decrease in Apo A-IV, in total amount of apolipoproteins adsorbed and an increase in Ig D (the exception was Klimofol) and Apo A-I (the exception was Recofol) was observed. In general, in between the adsorption patterns of the different emulsions, no large differences were found for Propofol-Lupro, -Fresenius, -Parke, Disoprivan and Diprivan. These similar patterns might explain that in *in vivo* human studies, no differences in the pharmacokinetic profiles were found [122]. However, recently, the effect of different propofol emulsions showed conflicting results by the use of different vehicles [123]. However, a conclusive study on the correlation of protein absorption patterns and *in vivo* organ distribution is still missing.

#### 2.2.9. Effect of Age

Emulsions are meta stable systems. Thus, particle growth can occur over time if the system is not sufficiently stabilized. Furthermore, during the shelf life of parenteral emulsions, lysolecithin is forming. This results in free fatty acids, which localize in the interface and change its properties, e.g., charge (change in zeta potential [124]). Of course, consequently, this could lead to changes in the adsorption pattern and in the resulting *in vivo* fate. Lipofundin N 20% emulsions of different ages were investigated (*i.e.*, freshly prepared, 16 and 28 months of age) regarding physical stability, surface charge and protein adsorption pattern [125]. The emulsions investigated remained stable over the time of observation; only very slight increases in zeta potentials were observed. Also, the protein adsorption patterns of these Lipofundin N emulsions showed little difference. Hence, the emulsions show not only a physical stability, but are also “biologically” stable regarding a little impairment of the MPS. Similar results have been obtained by analyzing aged Lipofundin N 10%, Lipofundin MCT 10% and Lipofundin MCT 20% [105]. 

It should be noted that the composition of the adsorption patterns on Lipofundin emulsions analyzed by Schmidt [105,125] differed markedly from the data published by Harnisch and Müller before. This was the case despite that, according to Schmidt, the same protocol for sample preparation prior to 2-D PAGE was used. There were a few years between the analyses, and theoretically, it might have been that the composition of the trade products might have changed slightly within the regulatory limits. However, assuming slightly changed compositions of the products, it is highly unlikely—considering the results above—that this would have had such a marked effect on the adsorption patterns. Therefore, it is likely that the centrifugation times were kept identical, but that the processing times of the samples in the lab were different (*cf*. Section 1.3). Due to the kinetics of protein adsorption, changes in the adsorption patterns still occur when the sample is standing around and during at least the first washing step, with some remaining plasma from the centrifugation pellet being present. These differences in the results show that a better standardization of sample preparation procedure is essential to obtain results which can be compared and clearly interpreted. A clear indication that the analysis is reproducible when performed each time the same way standardized (in this case by the operator Schmidt) is proven by the identical data from differently aged emulsions.

**Figure 8:**
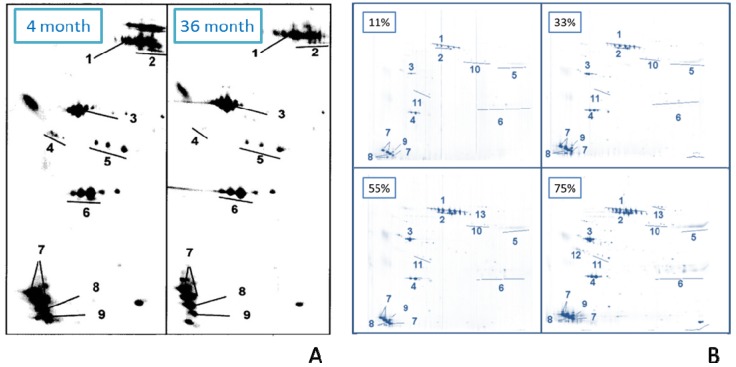
Influence of age and incubation time on adsorption patterns. (**A**) Close-ups of 2-DE gels four months stored and 26 months stored propofol loaded emulsion showing the lower left part, ranging from isoelectric point 4.4 (left gel side) to 5.7 (right gel side) and molecular weight 200 kD (upper gel area) to 6 kD (lower gel area). Protein spot number: (1) IgD, (2) Albumin, (3) ApoA-IV, (4) ApoJ, (5) ApoE, (6) ApoA-I, (7) ApoC-III, (8) ApoC-II, (9) ApoA-II (modified after [126]);(**B**) Protein adsorption pattern of Lipofundin MCT 20% oil-in-water emulsion incubated with different plasma concentrations (11%, 33%, 55%, 75%) (modified after [79]). Protein spot number: (1) Immunoglobulin D chain, (2) Albumin, (3) Apolipoprotein (Apo) A-IV, (4) Apo A-I, (5) Immunoglobulin g heavy chains, (6) Immunoglobulin light chains, (7) Apo C-III, (8) Apo C-II, (9) Apo A-II fragment, (10) Apo H, (11) Apo E, (12) Apo J, (13) Complement factor C3 (b-chain).

An identical study was performed with propofol-loaded emulsions [126]. The storage times were four and 26 months, respectively. Again, there was little change in the adsorption patterns. Figure 8A shows a close up of 2-DE gels of the two emulsions. From the data, it can be summarized that physically stable emulsions are also biologically stable; hence, the organ distribution upon IV administration is independent of the age of an emulsion.

#### 2.2.10. Adsorption Kinetics (Effect of Incubation Time)

The body distribution of intravenously injected drug carriers takes place very fast. Thus, it is important to collect information about the proteins adsorbing within the first seconds or minutes on the nanoemulsions. According to the “Vroman effect”, proteins with a high mobility and a large quantity in the blood will adsorb to the surface first, later being displaced by less abundant proteins with a higher affinity to the investigated surface [92,93].

Harnisch *et al.* examined the adsorption kinetics on emulsions and compared the obtained protein patterns with those previously gained from polystyrene particles of similar size [79]. Different concentrations of human plasma in the incubation medium have been used to prolong the residence time of more abundant plasma proteins on the surface of the emulsion droplets. In this way, proteins that are likely to be displaced in a split second are accessible to analysis. The nanoemulsion that has been used for the kinetic studies was the commercially available Lipofundin MCT 20%. A suspension of polystyrene particles (aqueous suspension, 2.69% (*w*/*v*) solid content) was the system with which to compare. Of course, it is not possible to perform a protein analysis in time intervals of a few seconds/minutes in which the Vroman effect takes place. An elegant solution to slow down adsorption/desorption is to incubate the samples in highly diluted plasma solutions with increasing concentrations (e.g., in this study with 11%, 33%, 55% and 75% plasma concentrations, respectively, Figure 8B). If no Vroman effect takes place, no differences between the different gels can be obtained, e.g., no protein desorption over time has been observed. Unlike the adsorption kinetics of solid polymeric particles, no ‘Vroman effect’ was observed for the nanoemulsion. However, the amount of the major proteins, mainly apolipoproteins, increased steadily with rising plasma concentrations (Figure 8B). A possible explanation for the different adsorption kinetics can be found in the different nature of the systems and, thereby, the different binding facilities of the proteins to the surface. The missing “Vroman effect” might be an advantage of the nanoemulsions as a stable, less time-dependent adsorption pattern that allows better utilization for drug targeting than a drug delivery system with an adsorption pattern being very dependent on contact time with proteins.

Looking more closely at the emulsion data in this study, a time-dependent kinetic cannot be excluded. A proof for the Vroman effect is when certain proteins increase in the amount adsorbed, with increasing plasma concentration in the incubation medium and then show, again, a decrease. In the case of the emulsion, there was only a continuous increase for the major proteins. However, it cannot be excluded that in the case of emulsions, the kinetics are slower; therefore, no effect was observed in this model, simulating the very first seconds and 1–2 min of adsorption. By now, no studies are published looking at longer incubation times, e.g., a few hours. The presence of, at least, slow kinetics is supported by the differences in the protein adsorption patterns of Lipofundin emulsions reported by Harnisch and Schmidt (*cf.* Section 2.2.10), which was attributed to different processing times and the resulting different times of contact between emulsions and plasma proteins.

For better resolution of the kinetics, it might also be better to replace the time-consuming centrifugation for separation of the droplets from the plasma by a faster method, e.g., chromatography with a small bed volume column, allowing separation within minutes. Even when incubating the sample only for 5 min, during centrifugation for 1 h, adsorption continues. Actually, the obtained pattern does not correspond to a 5 min pattern, but rather a 1 h 5 min pattern. 

## 3. Conclusions

The detection and use of protein adsorption patterns is relatively well-established for polymeric nanoparticles. For example, they could be exploited to identify the targeting moiety—Apo E—for directing nanoparticles to the brain, e.g., with dalargin. The validity could be proven by attaching Apo E prior to injection to the surface of the negative control, in this study, to non-brain specific nanoparticles. After the attachment of Apo E, [127] delivered the drug to the brain. The details of the preparation of the particles and their physico-chemical characteristics were known. There was also full information about their organ distribution. Hence, the concept of differential protein adsorption (Figure 1) could be confirmed, as the correlation between physico-chemical properties, acquired adsorption patterns and resulting organ distribution could be established.

Also, for nanoemulsions, the detection of the protein adsorption pattern is established and 2-D PAGE nicely proved its power in detecting differences between different emulsions, e.g., unloaded and drug-loaded nanoemulsions. Studies with commercial nanoemulsions provided basic information about the effect of the oil (inner core), the stabilizers and surface charge on the acquired adsorption pattern. Stabilizers were identified as the major players determining these patterns. 

It could also be proven that incorporation of amphiphilic drugs localizing in the interfaces and changing their composition can distinctly affect the adsorption patterns (e.g., amphotericin B). Differences were also detected for commercial propofol emulsions despite that they were derived from the same base nanoemulsion (Intralipid). However, by now, it is not possible to explain these differences (correlation of properties to patterns, Figure 1), first, because there is no information about the process differences applied by the manufacturers and, second, because there is a lack of data about the *in vivo* organ distributions of these different commercial products. 

However, this knowledge can be exploited for the controlled development of new nanoemulsion formulations. It is predictable that little changes will lead to a change in the protein adsorption pattern and, thus, to a different organ distribution. With that, the pharmacokinetic parameters (e.g., *c*_max_, *t*_max_) can change and, thus, the pharmacokinetic effects of the emulsion, e.g., increased or decreased treatment efficiency and/or toxicity. 

By now, in controlled academic studies, it was also demonstrated that the adsorption patterns can be easily modified, e.g., by simple dissolution of stabilizers in an existing emulsion, e.g., poloxamer 188. The stabilizers partition between water and interface localize automatically in the interface and change the properties of the emulsion droplets. In the case of poloxamer 188, the lecithin stabilized emulsion turned into an emulsion with a stealth-like adsorption pattern, being in agreement with the prolonged circulation times observed for poloxamer 188 emulsions [111]. Therefore, the combination of analysis of the adsorption patterns and the controlled modification of the surface properties by stabilizer admixture can be a useful tool to design emulsions optimized for *in vivo*, e.g., no adsorption of opsonins to avoid MPS impairment.

At present, it can be summarized that there is relatively much knowledge about the composition or certain physical parameters of the emulsions and the related adsorption patterns. However, there is a lack of related *in vivo* data for a full interpretation of the observed adsorption patterns. The reason for this, as outlined above, is the 2-D PAGE analysis itself, as it is extremely time consuming, complex and costly, the *in vivo* studies being even more costly. Furthermore, it needs to be considered that the determination of the organ distribution studies is more difficult with fast degradable emulsion droplets than with relatively slowly degrading polymeric nanoparticles. Another obstacle is the very limited number of research groups being active in this field of protein adsorption patterns. This was again realized when searching the up-to-date literature for this review. Quite a number of studies were done, but many with just our laboratory. Therefore, the references might give a little bit of the impression of quite a lot of self-citations. All this goes back to the fact that the role of the adsorbed proteins for the organ distribution has been recognized for more than 20 years, but has still not yet fully been reflected in intense research activities in the area of IV nanoparticles, especially not for IV nanoemulsions.

Obviously by now rather the traditional empirical approach is being taken: make the nanoemulsion and see how it behaves *in vivo*. For the future, a more rapid and target-directed development would be possible by analyzing the adsorbed proteins during formulation development. Controlling the key factor for organ distribution means shorter development times and a higher success rate to the market, not only for emulsions with NCEs, but also for generic products. Improving the organ distribution and pharmacokinetics of nanoemulsions can lead to super generics or new, more efficient, drug formulations on the market.

## Supplementary Files

Supplementary File 1 Supplementary Information (PDF, 726 KB)
