# Supplementary material for: Protein Adsorption Patterns and Analysis on IV Nanoemulsions—The Key Factor Determining the Organ Distribution
Source: Pharmaceutics. 2012 Dec 21;5(1):36–68. doi: 10.3390/pharmaceutics5010036 (PMC3834939; doi:10.3390/pharmaceutics5010036)
Supplement: Supplementary File 1 — Supplementary Information (PDF, 726 KB) [file pharmaceutics-05-00036-s001.pdf]

Supplementary Information

Table S1. Overview of the topics discussed, e.g., composition and size of the emulsions discussed.

Section	Study and characteristic parameters of the emulsions investigated
2.2.1	Adsorption patterns of 20% emulsions stabilized with lecithin from different manufactures
	Lipofundin MCT 20% (B. Braun Melsungen AG, Germany), Intralipid 20% (Pharmacia AB, Sweden), Abbolipid 20% (Abbott, Germany) and Schwalipid 20% were compared to each other. For all investigated fat emulsions the amount of stabilizer (lecithin) was about 1.2% and the particle size distribution of all these emulsions differed just slightly (mean diameter about 250 nm).
2.2.2 +	Adsorption patterns of different oil compositions & concentrations: LCT *vs*. LCT/MCT & 10% *vs*. 20%
2.2.3	Investigated were Lipofundin MCT 10%, Lipofundin N 10%, Lipofundin MCT 20% and Lipofundin N 20%. Lipofundin N emulsions consist of soy oil (LCT—long chain triglycerides), Lipofundin MCT emulsions are a mixture of 50:50 LCT and MCT. All the investigated emulsions were stabilized with lecithin (soy lecithin in case of Lipofundin N, egg lecithin in case of MCT emulsions). The 10% emulsions contained 0.8% lecithin, 20% emulsions contained 1.2%. Emulsions were typically in the range of 250–270 nm, only the 20% Lipofundin N emulsion was about 350 nm.
2.2.4	Effect of type of oil phase on protein adsorption
	So called “structured lipids” (SLs) as alternative to LCT in emulsions for parenteral nutrition were investigated and compared to an LCT emulsion (Intralipid N 10%). The emulsions investigated were composed of Short-Long-Short (SLS) fatty acids at the glycerol back bone, the respective exchange with medium chain fatty acids (MLM SLs) and long fatty acids (LLL) (e.g., pure soy bean oil). The overall composition of the emulsions was: 10% lipid phase, 1.2% Lipoid E80 as emulsifier and 2.1% glycerol. The average particle size was: 265 nm (Intralipid), 349 nm (LLL), 407 nm (MLM) and 306 nm (SLS).
2.2.5	Effect of stabilizer composition on adsorption patterns.
	The effect on fatty acids with different chain lengths and a non-ionic PEG containing stabilizer (Solutol) and mixtures of these stabilizers in comparison to a solely lecithin stabilized emulsion were investigated. The detailed formulations investigated in this study are shown in Table 4.
2.2.6	Surface modification of Lipofundin emulsions for drug targeting
	Lipofundin MCT 10% without modification and Lipofundin MCT 10% upon admixing of 2% Poloxamer 407 solution were analysed and compared to each other.
2.2.7	Influence of surface charge
	Four different formulations (two anionic and two cationic emulsions) and the commercially available anionic emulsion Lipofundin MCT 10% were investigated. The standard composition consisted of MCT (8.5% *w*/*w*), Lipoid E-80 (1.2% *w*/*w*), α-tocopherol (0.02% *w*/*w*), Poloxamer 188 (2.0% *w*/*w*), glycerol (2.25% *w*/*w*) and bi-distilled water (to 100% *w*/*w*) with additional stearylamine (0.3% *w*/*w*) or oleylamine (0.3% *w*/*w*) for the two cationic formulations and oleic acid (2.83% *w*/*w*) or deoxycholic acid (0.5% *w*/*w*) for the two anionic formulations. The physical characterization of these nanoemulsions is given in Table 6.

Table S1. *Cont.*

Section	Study and characteristic parameters of the emulsions investigated
2.2.8	Effects of drug incorporation
	Effect of Amphotericin B: Amphotericin B was incorporated in the commercial fat emulsion Lipofundin N 20% applying the SolEmuls process [119,120]. The drug load was 1 mg/mL. Effect of Propofol: Eight different marketed propofol nanoemulsions were investigated and compared with the respective emulsion base Lipofundin MCT 10% and Intralipid. Table 9 gives an overview of the investigated emulsions, they were all in the size range of about 200–259 nm.
2.2.9	Effect of age
	Lipofundin N 10%, Lipofundin MCT 10%, and Lipofundin MCT 20% and Lipofundin N 20% emulsions of different age were investigated (*i.e.*, freshly prepared, 16 and 28 months of age) regarding size, physical stability and protein adsorption pattern. Furthermore, a study was performed with propofol-loaded emulsions. The storage times were 4 and 26 months, respectively.
2.2.10	Adsorption kinetics (effect of incubation time)
	The commercially available Lipofundin MCT 20% was used and incubated with increasing concentrations of plasma (11%, 33%, 55% and 75%, respectively).

Table S2. Amounts of major proteins as percentage of the overall detected amount of protein of different emulsions containing 20% lipid phase (after [103]).

plasma proteins	Lipofundin MCT 20%	Intralipid 20%	Abbolipid 20%	Schwalipid 20%
ApoA-I	24.0	20.9	24.0	22.4
ApoA-IV	19.5	19.9	19.8	20.1
ApoC-II	13.9	15.0	14.1	13.6
ApoC-III	1.3	1.4	1.2	1.7
IgD	29.4	30.2	28.6	27.5

Figure S1. Influence of oil composition on adsorption patterns. Upper: Lipofundin MCT 10% (**A**) and Lipofundin N 10%; (**B**) Lower: Lipofundin N 20%; (**C**) and Lipofundin MCT 20%; (**D**) Views are close-ups of the 2-DE gels, each showing the lower left part of the gels, ranging from isoelectric point 4.4 (left gel side) to 5.7 (right gel side) and molecular weight 200 kD (upper) to 6 kD (lower): Protein spot number: (1) IgD, (2) Albumin, (3) ApoA-IV, (4) ApoJ, (5) ApoE, (6) ApoA-I, (7) ApoC-III, (8) ApoC-II, (9) ApoA-II (modified after [105]).

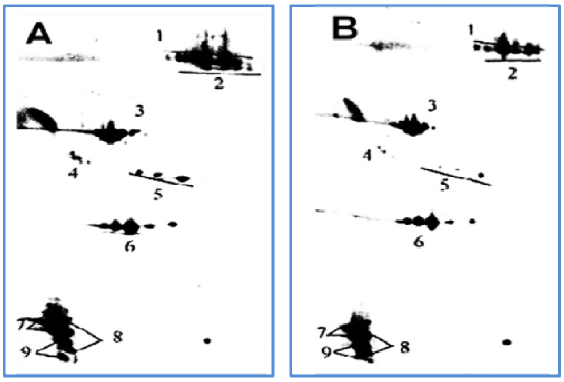

Figure S1. *Cont.*

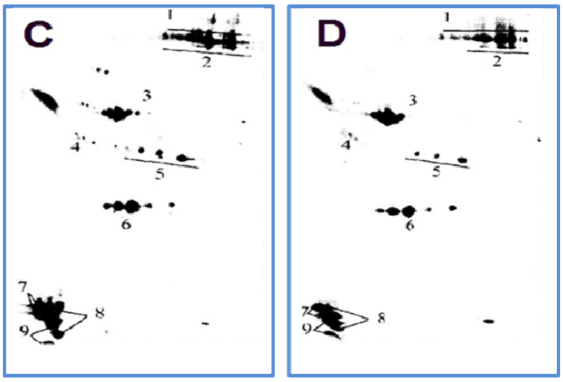

Figure S2. Schematic drawing of structured lipids (SLS, MLM) and a long-chain triglyceride (LLL). S is short-chain fatty acids (C_4), M is medium-chain fatty acids ($C_{9–10}$) and L represents long-chain fatty acids ($C_{16–18}$) (modified after [108]).

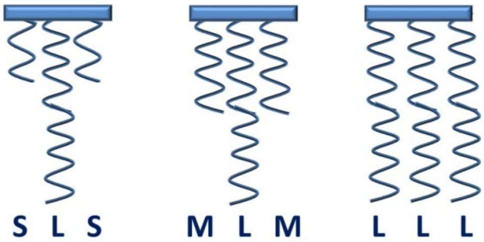

Table S3. Composition of the emulsifier blends of the investigated oil in water emulsions (20% lipid phase composed of peanut oil). Values as % (*w*/*w*) (after [110]).

Emulsifier blends	Phospholipon 80	Solutol	Na-Myristate	Na-Stearate
Phospholipon 80	1.5	-	-	-
Phospholipon 80 + Na-Myristate	1.5	-	0.25	-
Phospholipon 80 + Na-Stearate	1.5	-	-	0.15
Phospholipon 80 + Solutol	1.5	1.0	-	-
Phospholipon 80 + Solutol + Na-Myristate	1.5	1.0	0.15	-
Phospholipon 80 + Solutol + Na-Stearate	1.5	1.0	-	0.15

Table S4. Plasma protein adsorption (vol.%) on surfaces of Lipofundin N 20% and amphotericin B-containing fat emulsion based on Lipofundin N 20% (after [80]).

proteins	Lipofundin N 20%	Lipofundin N 20% + AmB
Albumin	6.0	3.4
ApoA-I	17.6	15.5
ApoA-IV	12.4	8.3
ApoC-III	16.8	8.7
ApoJ	2.5	5.9
Fibrinogen-α	0	1.2
Fibrinogen-β	0	2.0
Fibrinogen-γ	0.4	3.9

Table S5. Propofol loaded emulsions with manufacturer name, batch no. and type of oil component (after [77]).

Commercial product	Manufacturer	Batch no.	Oil component
Diprivan 1%	Zeneca	A 70478 A	LCT
Disoprivan 1%	Zeneca/Glaxo	4389 Y	LCT
Klimofol 1%	IV Amed	A70698 A	LCT
Propofol Abbott 1%	Abbott	31 989 Z 7	LCT
Propofol 1% Fresenius	Fresenius	HK 1614	LCT
Propofol-Lipuro 1%	B Braun Melsungen AG	6244 A 31	LCT/MCT
Propofol 1% Parke-Davis	Parke-Davis	97 D 16	LCT
Recofol	Leiras	714562	LCT
Lipofundin MCT 10%	B Braun Melsungen AG	6023A 81	LCT/MCT
Intralipid 10%	Pharmacia AB	73457-51	LCT

© 2013 by the authors; licensee MDPI, Basel, Switzerland. This article is an open access article distributed under the terms and conditions of the Creative Commons Attribution license (http://creativecommons.org/licenses/by/3.0/).
